# The efficacy of thuricin CD, tigecycline, vancomycin, teicoplanin, rifampicin and nitazoxanide, independently and in paired combinations against *Clostridium difficile* biofilms and planktonic cells

**DOI:** 10.1186/s13099-016-0102-8

**Published:** 2016-06-02

**Authors:** Harsh Mathur, Mary C. Rea, Paul D. Cotter, Colin Hill, R. Paul Ross

**Affiliations:** School of Microbiology, University College Cork, Cork, Ireland; Teagasc Food Research Centre, Moorepark, Fermoy, County Cork Ireland; Alimentary Pharmabiotic Centre Microbiome Institute, University College Cork, Cork, Ireland; College of Science, Engineering and Food Science, University College Cork, Cork, Ireland

**Keywords:** Biofilms, *Clostridium difficile*, Antimicrobial combinations, Tigecycline, Nitazoxanide, Rifampicin

## Abstract

**Background:**

Thuricin CD is a two-component antimicrobial, belonging to the recently designated sactibiotic subclass of bacteriocins. The aim of this study was to investigate the effects of thuricin CD, as well as the antibiotics, tigecycline, vancomycin, teicoplanin, rifampicin and nitazoxanide when used independently and when combined at low concentrations on the viability of *Clostridium difficile* 20291 R027, TL178 R002, Liv022 R106, DPC6350 and VPI10463 biofilms and planktonic cells.

**Results:**

On the basis of XTT (2,3-bis[2-methyloxy-4-nitro-5-sulphophenyl]-2H-tetrazolium-5-carboxanilide)-menadione biofilm viability assays, we found that thuricin CD was effective against biofilms of R027, Liv022 R106 and DPC6350 when used independently while nitazoxanide and rifampicin were also potent against biofilms of R027 and DPC6350, when applied on their own. Tigecycline was found to be effective against R027 and DPC6350 biofilms, whereas teicoplanin and vancomycin when used independently were only effective against DPC6350 biofilms. The efficacies of the antibiotics rifampicin, tigecycline, vancomycin and teicoplanin against *C. difficile* 20291 R027 biofilms were significantly potentiated when combined with thuricin CD, indicating effective antimicrobial combinations with this sactibiotic against R027 biofilms. However, the potency of nitazoxanide against R027 biofilms was significantly diminished when combined with thuricin CD, indicating an ineffective combination with this sactibiotic against R027 biofilms. Paired combinations of thuricin CD along with these five antibiotics were effective at diminishing the viability of DPC6350 biofilms. However, such combinations were largely ineffective against biofilms of TL178 R002, Liv022 R106 and VPI10463.

**Conclusions:**

To the best of our knowledge, this is the first study to highlight the activity of a sactibiotic bacteriocin against biofilms and the first to reveal the potency of the antibiotics tigecycline, teicoplanin and nitazoxanide against *C. difficile* biofilms. On the basis of this study, it is apparent that different strains of *C. difficile* possess varying abilities to form biofilms and that the sensitivities of these biofilms to different antimicrobials and antimicrobial combinations are strain-dependent. Since the formation of relatively strong biofilms by certain *C. difficile* strains may contribute to increased cases of antibiotic resistance and recurrence and relapse of *C. difficile* infection, the findings presented in this study could provide alternative strategies to target this pathogen.

## Background

*Clostridium difficile* infection (CDI) is a major healthcare concern, especially in the European Union and North America [1]. In recent years, the formation of biofilms by *C. difficile*, which could contribute to antibiotic resistance and CDI treatment failures has been highlighted [2, 3]. Indeed, increased resistance of *C. difficile* to vancomycin and metronidazole and recurrence of disease due to treatment failure have already been reported [4]. Thus, there is a growing need to find alternative solutions to combat this pathogen. Two of the most problematic *C. difficile* biofilm producers are strains 20291 R027 and strain 630 [5]. Recent studies have provided insights into the mechanisms by which *C. difficile* forms biofilms [2, 3, 5]. Dawson et al. demonstrated that the ability of *C. difficile* to produce biofilms involves the formation of several layers of bacteria in a complex matrix which is composed of polysaccharides, proteins and DNA [2]. Furthermore, Dapa et al. showed that a quorum sensing regulator, LuxS, flagellae and Cwp84 are all needed for optimal *C. difficile* biofilm formation [5]. The authors of the study also found that there could be a potential link between the ability to form biofilms and sporulation, as *C. difficile* strains with a mutation in the gene encoding SpoOA, which is a transcription factor needed for sporulation, lacked the ability to form biofilms [5].

In this study, we provide insights into the potencies of various antimicrobials and antimicrobial combinations, when combined at relatively low concentrations against various *C. difficile* biofilms and compare and contrast the effects of these combinations against planktonic cells of the same strains. We note that thuricin CD is effective at targeting the biofilms of R027, R106 and DPC6350 at relatively low concentrations, while paired combinations of this bacteriocin with the antibiotics teicoplanin, tigecycline, vancomycin or rifampicin are potent at attenuating the viability of adherent biofilms formed by *C. difficile* strains 20291 R027 and DPC6350.

## Methods

### Thuricin CD purification

Thuricin CD was purified as described by Rea et al. with minor modifications [6]. Briefly, brain heart infusion (BHI) broth was first clarified using XAD-16 beads (Sigma Aldrich) prior to autoclaving. The thuricin CD-producing strain *Bacillus thuringiensis* DPC6431 was subcultured two times in BHI broth prior to inoculating 1.5 L of clarified BHI broth and grown overnight at 37º C with vigorous agitation. The overnight culture was centrifuged at 8260*g* for 20 min and both the supernatant and cell pellet were kept for further use. The cell pellet was resuspended in 300 ml of 70 % isopropanol (IPA), 0.1 % TFA and stirred for 4 h at 4 ºC. The supernatant was passed through fresh XAD-16 beads in a column and subsequently washed with 500 ml of 35 % ethanol. Thuricin CD was eluted in 400 ml of 70 % IPA, 0.1 % TFA and this elute was called S1. The cell pellet which was resuspended in 70 % IPA, 0.1 % TFA was centrifuged at 8260*g* for 20 min and the supernatant (S2) was combined with S1. All the IPA was removed by using rotary evaporation (Buchi) and the sample passed through a Phenomenex C-18 column which had been pre-equilibrated with methanol and water. The column was washed with 120 ml of 35 % ethanol and thuricin CD eluted in 75 ml of 70 % IPA, 0.1 % TFA. This was further concentrated by removing the IPA using rotary evaporation, prior to separating the two peptides using reverse-phase high performance liquid chromatography (RP-HPLC).

### Minimum inhibitory concentrations (MICs) determinations

The antibiotics rifampicin, teicoplanin, tigecycline, vancomycin and nitazoxanide for this study were purchased from Sigma Aldrich. The MICs of the sactibiotic, thuricin CD and the antibiotic vancomycin against *C. difficile* strains 20291 R027, TL178 R002 and Liv022 R106 had already been determined in our previous study [7]. MICs of the antibiotics teicoplanin, tigecycline, rifampicin and nitazoxanide against planktonic cells of *C. difficile* strains 20291 R027, Liv022 R106, TL178 R002, DPC6350 and VPI10463 were determined in this study and all assays were conducted as described in Mathur et al. [7]. Briefly, reinforced clostridium medium (RCM), previously boiled and cooled under anaerobic conditions, was used for overnight cultures of the various *C. difficile* strains, grown at 37 °C in an anaerobic chamber for 16 h. The antibiotics were weighed out and resuspended initially in RCM broth (for teicoplanin, tigecycline, vancomycin and rifampicin) and DMSO for nitazoxanide. Antibiotics were subsequently diluted in RCM broth to give the desired starting concentrations and serially diluted in 96-well microtitre plates. Overnight cultures of *C. difficile* strains were subcultured until they reached mid-logarithmic phase (OD_600_ of approximately 0.5), at which point they were inoculated into the wells of the microtitre plates, with a final inoculum of approximately 5 × 10^5^ cfu/ml. MIC readings were taken at 18 h and the MIC was defined as the lowest concentration of the antibiotic at which there was no visible growth. Assays were conducted in triplicate.

### Fractional inhibitory concentration (FIC) determinations

FIC values were determined by conducting broth microdilution checkerboard assays, as described by Orhan et al. [8]. Briefly, two antimicrobials were combined on the same microtitre plate, such that antimicrobial A was serially diluted vertically along the microtitre plate and antimicrobial B serially diluted from right to left horizontally along the microtitre plate, resulting in a mix of the two antimicrobials at different concentrations in different wells in the same microtitre plate. The plates were inoculated with approximately 5 × 10^5^ cfu/ml, as described for the MIC assays and FIC indices determined after 18 h. Assays were conducted in triplicate. The antimicrobial interactions and FIC values were interpreted as synergistic, partial synergistic, additive, indifferent or antagonistic, as described by Orhan et al. and Bacon et al. [8, 9]. FIC values for thuricin CD-vancomycin combinations against R027, Liv022 R106 and TL178 R002 were determined and reported in our previous study [7].

### XTT assays to determine the efficacy of antimicrobials and antimicrobial combinations against *C. difficile* biofilms

The activities of the above-mentioned antimicrobials were also assessed against *C. difficile* 20291 R027, Liv022 R106, TL178 R002, DPC6350 and VPI10463 biofilms using the XTT (2-methyloxy-4-nitro-5-sulfophenyl]-2H-tetrazolium-5-carboxanilide)-menadione reduction assay, as described by Field et al. with minor modifications [10]. Briefly, *C. difficile* biofilms on 96-well microtitre plates were formed by incubating approximately 5 × 10^5^ cfu/ml of log-phase cells in BHI broth supplemented with 0.1 M glucose (for R027, Liv022 R106 and TL178 R002) or RCM broth supplemented with 0.1 M glucose (for DPC6350 and VPI10463) for 72 h in an anaerobic workstation. After 72 h, the supernatants were removed from the wells and the biofilms washed very gently with phosphate buffered saline (PBS) to eliminate planktonic cells. Antimicrobials and antimicrobial combinations resuspended in BHI broth supplemented with 0.1 M glucose (or RCM broth supplemented with 0.1 M glucose for strains DPC6350 and VPI10463) were added to the wells and allowed to incubate for 24 h under anaerobic conditions. After 24 h, the supernatants were removed and the biofilms washed extremely gently with PBS. At this point, 100 µl of XTT was added to each well and incubated in the dark under anaerobic conditions for 2 h. After 2 h, OD_492_ readings were taken using a plate reader (BioTek Synergy HT, Vermont, USA). At least five independent replicates were used for each strain and each condition tested. Statistically significant differences between untreated and treated samples were determined using Student’s *t* test.

## Results

### Minimum inhibitory concentrations (MICs) of various antimicrobials against *C. difficile* strains

The MIC range for thuricin CD against the *C. difficile* strains used in this study was 0.703–2.812 µg/ml (125–500 nM), whereas the MICs for vancomycin against the *C. difficile* strains ranged between 0.464–1.856 µg/ml (312–1250 nM)(Table 1). In terms of molar concentrations, it was found that rifampicin was consistently the most potent antimicrobial tested against the *C. difficile* strains tested in this study, with MIC values in the low nanomolar range (values ranged between 0.0016–0.0064 µg/ml)(Table 1). Rifampicin MICs determined by the E test were found to be between 0.002–32 μg/ml in a study conducted by O’Connor et al. while Huhulescu and co-workers reported rifampicin MIC values ranging from ≤0.002 to ≥32 µg/ml against a variety of *C. difficile* isolates, also based on E tests [11, 12]. Tigecycline and teicoplanin were found to have largely similar MIC values against *C. difficile* in this study, with tigecycline values ranging from 0.091 to 0.366 µg/ml and teicoplanin values ranging between 0.146 and 0.292 µg/ml (Table 1). The MIC_90_ range for tigecycline against *C. difficile* has been reported to be between 0.03 and 0.25 μg/ml in other studies [13, 14]. The MICs of teicoplanin against *C. difficile* were between 0.023 and 0.75 μg/ml in previous studies, whereas de Lalla and co-workers documented MIC_50_ and MIC_90_ values of teicoplanin against *C. difficile* to be less than 0.125–0.25 μg/ml [15–17]. In terms of molar values, nitazoxanide was consistently the least potent antimicrobial against *C. difficile* strains in this study, with the MIC value range between 390 and 1560 nM (0.12–0.479 µg/ml). The MIC range for nitazoxanide against *C. difficile* was found to be between 0.03 and 1.0 µg/ml in other studies [13, 18]. As mentioned previously, thuricin CD and vancomycin display potent anti-*C. difficile* activity in terms of molar MIC concentrations. However, vancomycin and, thuricin CD in particular (due to its two-component nature and consequent high molecular weight), appear the least potent antimicrobials against *C. difficile* when MIC values are expressed in µg/ml (Table 1).

**Table 1:** Minimum inhibitory concentration (MIC) values of antimicrobials against *C. difficile* strains

Antimicrobial/Strain	Teicoplanin nM (μg/ml)	Tigecycline nM (μg/ml)	Nitazoxanide nM (μg/ml)	Rifampicin nM (μg/ml)	Thuricin CD^a^ nM (μg/ml)	Vancomycin^b^ nM (μg/ml)
20291 R027	78 *(0.146)*	312 *(0.183)*	1560 *(0.479)*	8 *(0.0064)*	125 *(0.703)*	625 *(0.928)*
Liv022 R106	156 *(0.292)*	625 *(0.366)*	780 *(0.239)*	2 *(0.0016)*	250 *(1.406)*	1250 *(1.856)*
TL178 R002	156 *(0.292)*	625 *(0.366)*	390 *(0.120)*	2 *(0.0016)*	500 *(2.812)*	625 *(0.928)*
DPC6350	156 *(0.292)*	156 *(0.091)*	1560 *(0.479)*	2 *(0.0016)*	125 *(0.703)*	625 *(0.928)*
VPI10463	78 *(0.146)*	156 *(0.091)*	780 *(0.239)*	2 *(0.0016)*	125 *(0.703)*	312 *(0.464)*

MICs of various antimicrobials against planktonic cells of *C. difficile* strains expressed in nanomolar concentrations and also in μg/ml in parentheses and in italics

The MICs of thuricin CD^a^ and vancomycin^b^ against strains 20291 R027, Liv022 R106 and TL178 R002 were already determined and reported in our previous study [7]

### Determination of fractional inhibitory concentration (FIC) indices of various antimicrobial combinations against *C. difficile* strains

Fractional inhibitory concentrations (FIC) values were also determined by using broth microdilution checkerboard assays in this study, as described in Orhan et al. [8], and FIC values and effects interpreted as described in Bacon et al. [9] (Table 2). Merely partial synergistic (0.5 ≤ FIC ≤ 0.75), additive (0.75 ≤ FIC ≤ 1.0) and indifferent effects (1.01 ≤ FIC ≤ 2.0) were obtained in this study and no antagonistic effects (FIC ≥ 2.0) were observed. It should be emphasised, however, that checkerboard assays referred to here were conducted with planktonic *C. difficile* cells, to facilitate a comparison between these results and those with *C. difficile* biofilms described below.

**Table 2:** Fractional inhibitory concentration (FIC) values of various antimicrobial combinations against *C. difficile*

Antimicrobial combination/*C. difficile* strain	TCD-Teico^a^ ΣFIC	TCD-Tig^b^ ΣFIC	TCD-Rif^c^ ΣFIC	TCD-Nitaz^d^ ΣFIC	TCD-V^e, i^ ΣFIC
20291 R027	0.75 (PS)^f^	1.0 (Ad)^g^	1.0 (Ad)	1.01–2 (I)^h^	1.01–2 (I)
Liv022 R106	1.01–2 (I)	1.01–2 (I)	1.01–2 (I)	1.01–2 (I)	1.0 (Ad)
TL178 R002	1.01–2 (I)	1.01–2 (I)	1.01–2 (I)	1.0 (Ad)	1.01–2 (I)
DPC6350	1.01–2 (I)	1.01–2 (I)	1.01–2 (I)	1.0 (Ad)	1.01–2 (I)
VPI10463	1.01–2 (I)	1.01–2 (I)	1.01–2 (I)	1.0 (Ad)	1.01–2 (I)

Antimicrobial combination effects are indicated in parentheses

*PS* partial synergy (0.5 ≤ FIC ≤ 0.75), *Ad* additive effects (0.75 ≤ FIC ≤ 1.0), *I* indifferent effects (1.01 ≤ FIC ≤ 2.0)

FIC indices of ^a^thuricin CD-teicoplanin, ^b^thuricin CD-tigecycline, ^c^thuricin CD-rifampicin ^d^thuricin CD-nitazoxanide and ^e^thuricin CD-vancomycin against *C. difficile* strains. ^f^Thuricin CD-vancomycin FIC values against strains 20291 R027, Liv022 R106 and TL178 R002 were already determined and reported in our previous study [7]

### Efficacy of antimicrobials independently against *C. difficile* biofilms

**Fig. 1:**
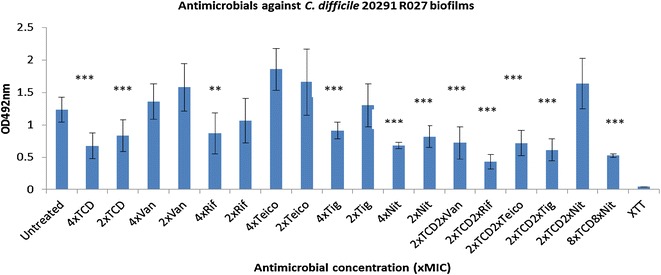
Activity of antimicrobials independently, and in combination with thuricin CD, against *C. difficile* 20291 R027 biofilms. Effects of various antimicrobials on the viability of *C. difficile* 20291 R027 biofilms, using the XTT-menadione viability assay assessed by measuring OD_492_ values. Antimicrobials were used at 4x and 2x MIC as well as combinations of 2x MIC thuricin CD with each of the antibiotics at 2x MIC. *Asterisks* indicate statistically significant reductions in biofilm viabilty relative to the untreated control, determined using the Student’s t test; *P < 0.05, **P < 0.01, ***P < 0.001. *TCD* thuricin CD, *Van* vancomycin, *Rif* rifampicin, *Teico* teicoplanin, *Tig* tigecycline, *Nit* nitazoxanide

**Fig. 2:**
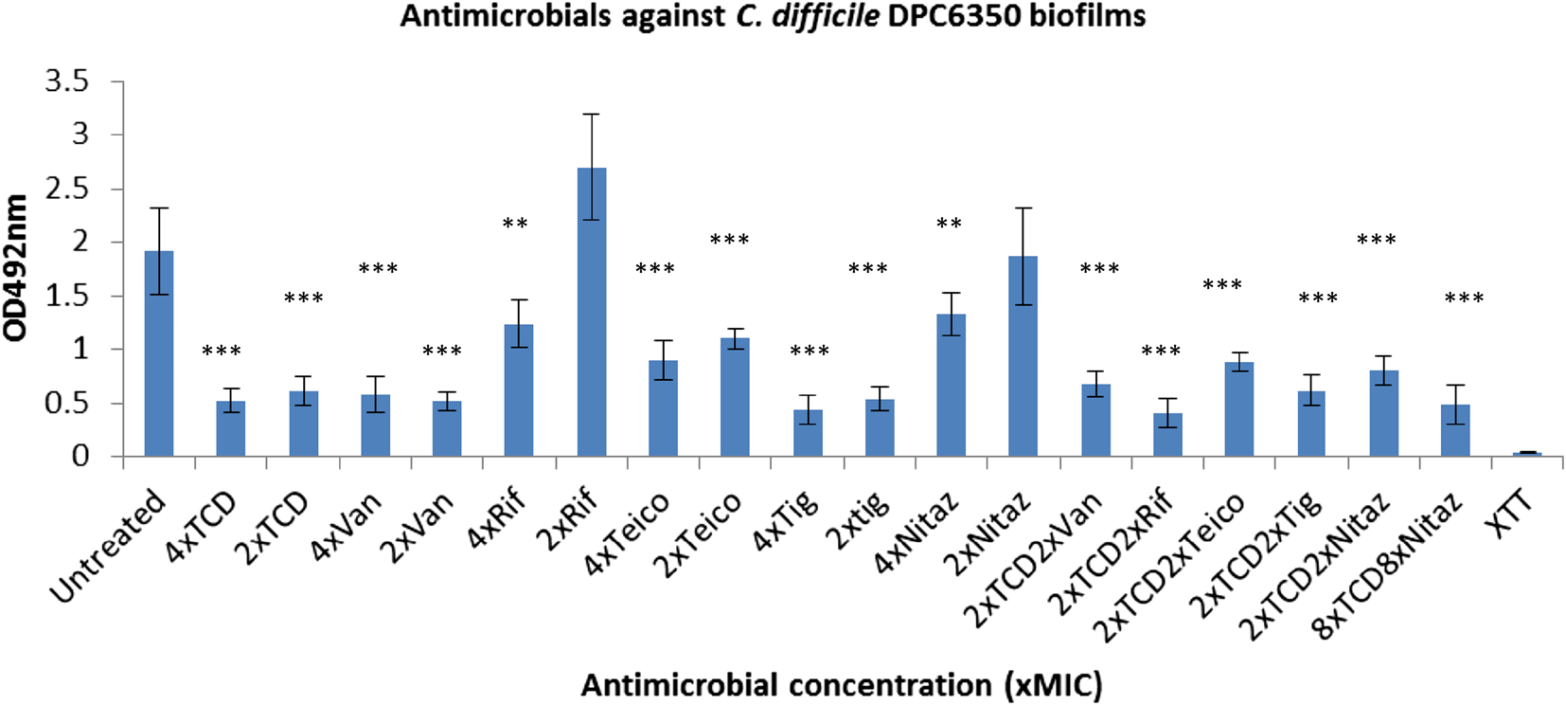
Activity of antimicrobials independently, and in combination with thuricin CD, against *C. difficile* DPC6350 biofilms. Effects of various antimicrobials on the viability of *C. difficile* DPC6350 biofilms, using the XTT-menadione viability assay assessed by measuring OD_492_ values. Antimicrobials were used at 4x and 2x MIC as well as combinations of 2x MIC thuricin CD with each of the antibiotics at 2x MIC. *Asterisks* indicate statistically significant reductions in biofilm viabilty relative to the untreated control, determined using the Student’s t test; *P < 0.05, **P < 0.01, ***P < 0.001. *TCD* thuricin CD, *Van* vancomycin, *Rif* rifampicin, *Teico* teicoplanin, *Tig* tigecycline, *Nit* nitazoxanide

**Fig. 3:**
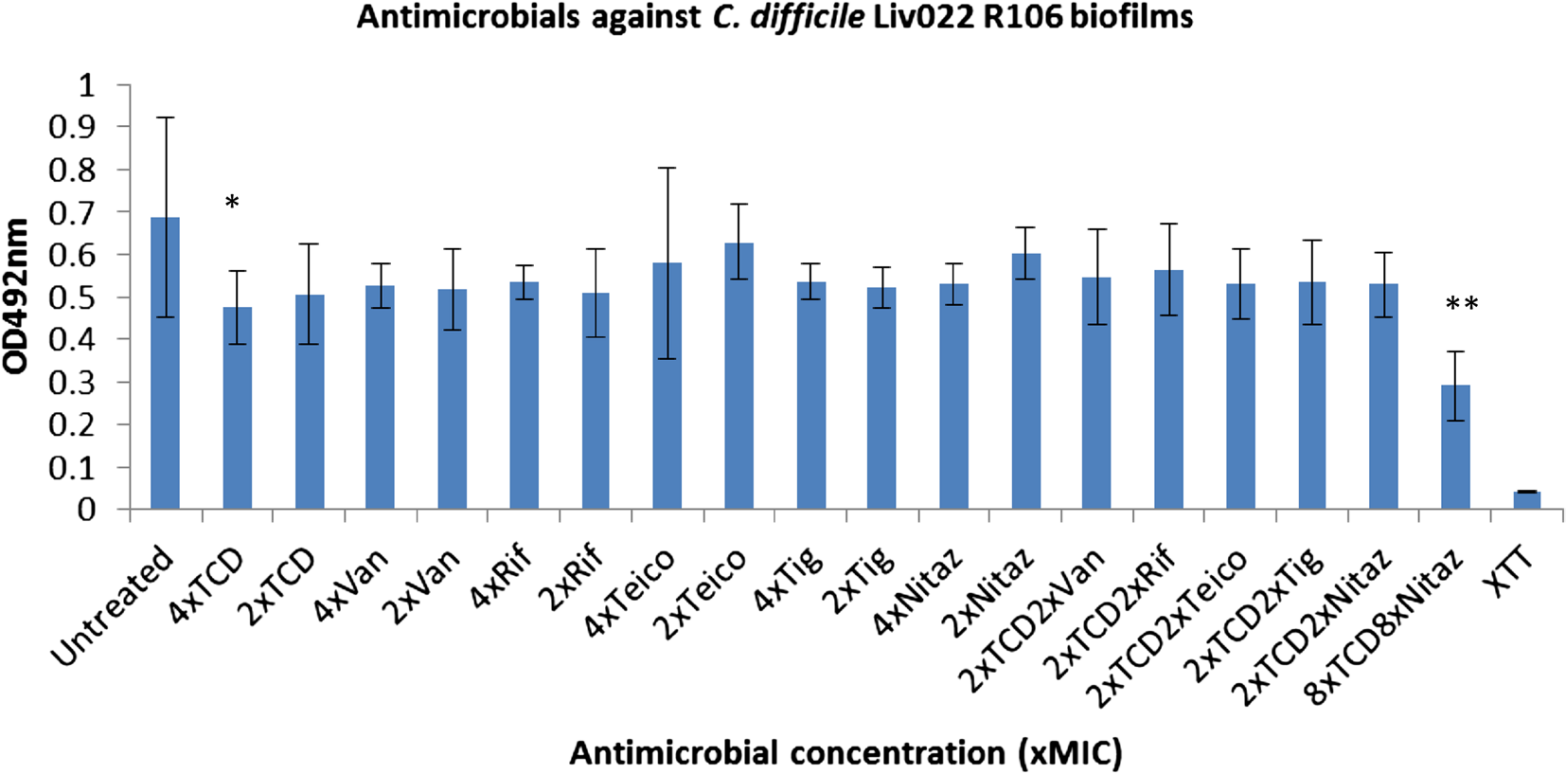
Activity of antimicrobials independently, and in combination with thuricin CD, against *C. difficile* Liv022 R106 biofilms. Effects of various antimicrobials on the viability of *C. difficile* Liv022 R106 biofilms, using the XTT-menadione viability assay assessed by measuring OD_492_ values. Antimicrobials were used at 4x and 2x MIC as well as combinations of 2x MIC thuricin CD with each of the antibiotics at 2x MIC. *Asterisks* indicate statistically significant reductions in biofilm viabilty relative to the untreated control, determined using the Student’s t test; *P < 0.05, **P < 0.01, ***P < 0.001. *TCD* thuricin CD, *Van* vancomycin, *Rif* rifampicin, *Teico* teicoplanin, *Tig* tigecycline, *Nit* nitazoxanide

**Fig. 4:**
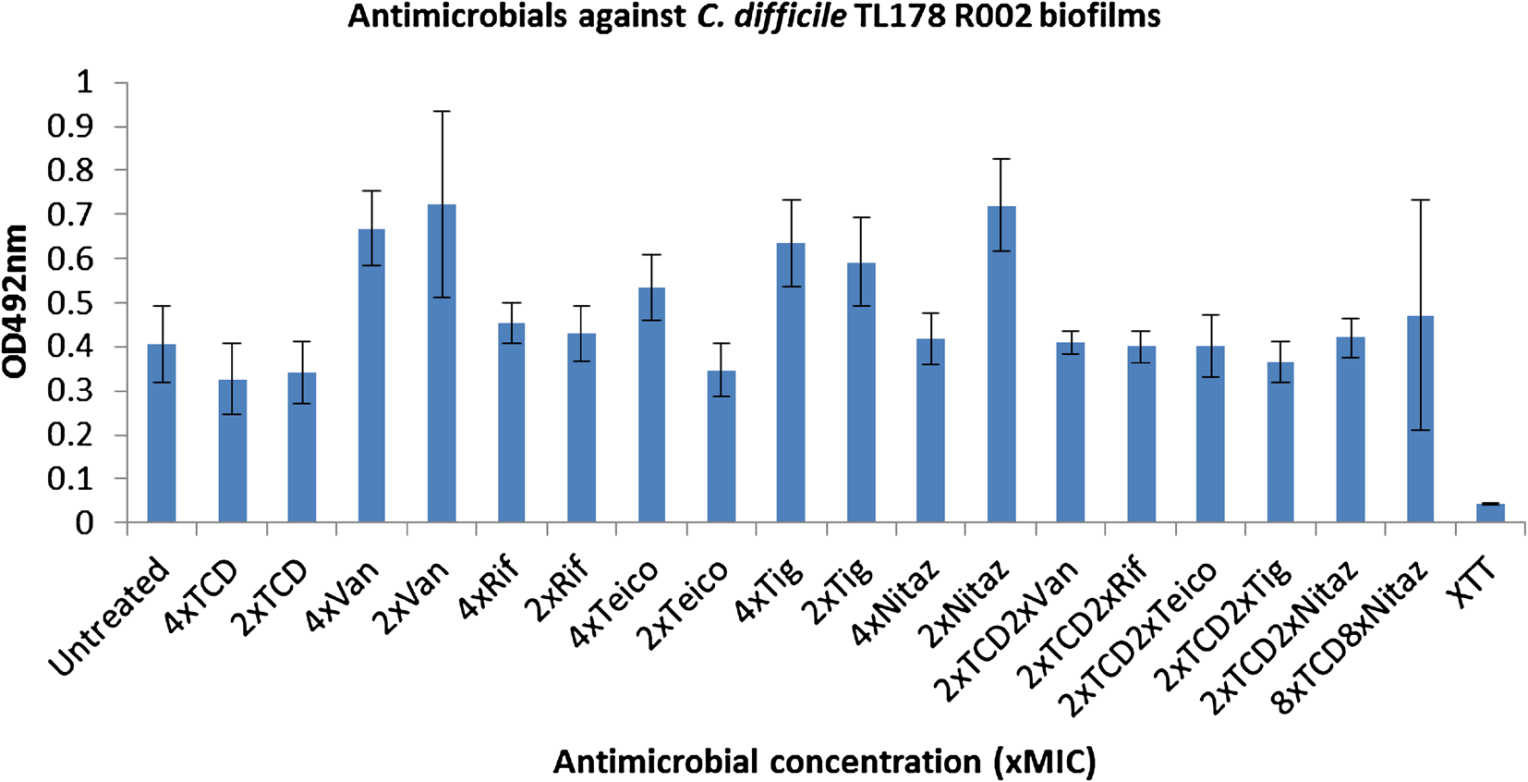
Activity of antimicrobials independently, and in combination with thuricin CD, against *C. difficile* TL178 R002 biofilms. Effects of various antimicrobials on the viability of *C. difficile* TL178 R002 biofilms, using the XTT-menadione viability assay assessed by measuring OD_492_ values. Antimicrobials were used at 4x and 2x MIC as well as combinations of 2x MIC thuricin CD with each of the antibiotics at 2x MIC. *Asterisks* indicate statistically significant reductions in biofilm viability relative to the untreated control, determined using the Student’s t test; *P < 0.05, **P < 0.01, ***P < 0.001. *TCD* thuricin CD, *Van* vancomycin, *Rif* rifampicin, *Teico* teicoplanin, *Tig* tigecycline, *Nit* nitazoxanide

**Fig. 5:**
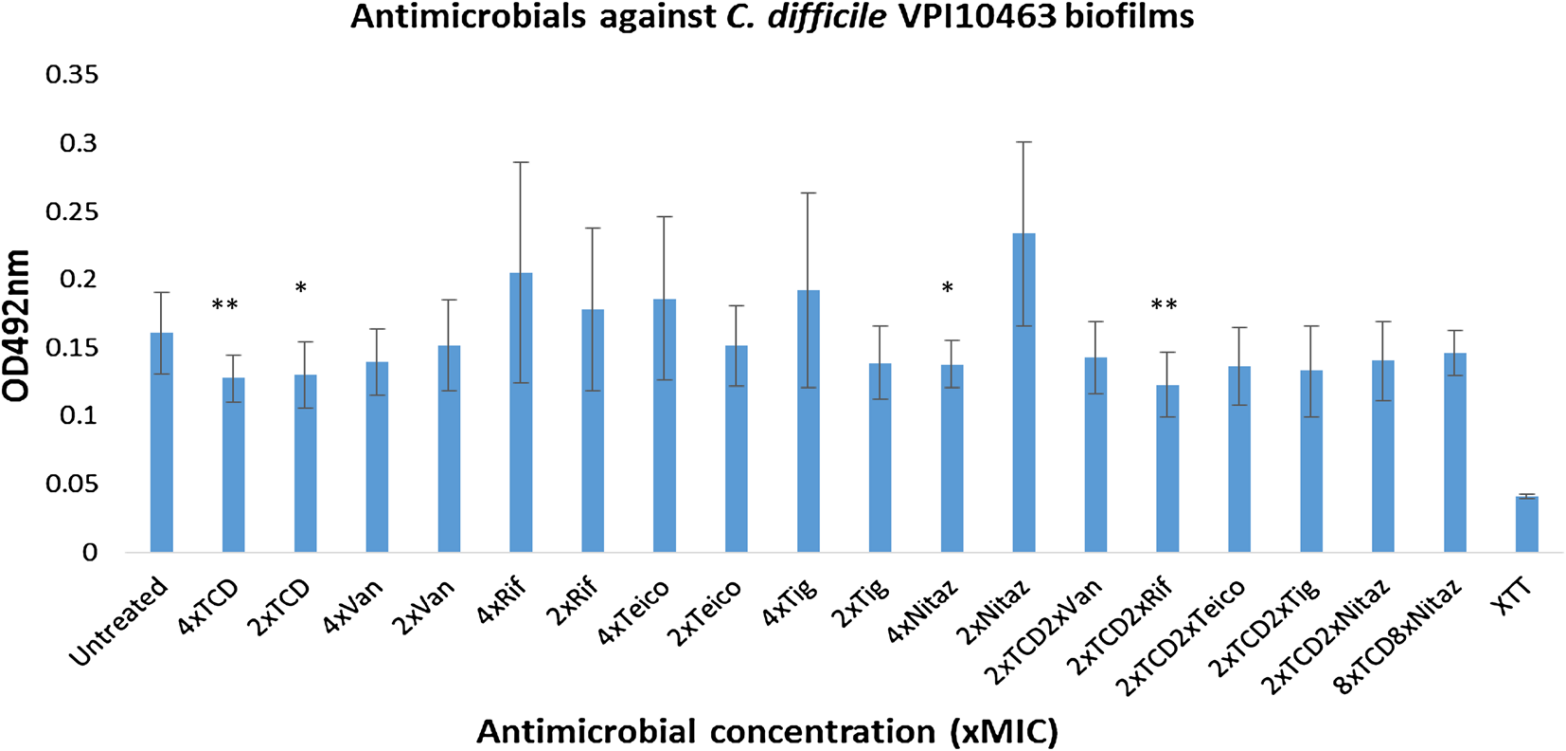
Activity of antimicrobials independently, and in combination with thuricin CD, against *C. difficile* VPI10463biofilms. Effects of various antimicrobials on the viability of *C. difficile* VPI10463 biofilms, using the XTT-menadione viability assay assessed by measuring OD_492_ values. Antimicrobials were used at 4x and 2x MIC as well as combinations of 2x MIC thuricin CD with each of the antibiotics at 2x MIC. *Asterisks* indicate statistically significant reductions in biofilm viabilty relative to the untreated control, determined using the Student’s t test; *P < 0.05, **P < 0.01, ***P < 0.001. *TCD* thuricin CD, *Van* vancomycin, *Rif* rifampicin, *Teico* teicoplanin, *Tig* tigecycline, *Nit* nitazoxanide

The activities of the above-mentioned antimicrobials were also assessed against *C. difficile* 20291 R027, TL178 R002, Liv022 R106, DPC6350 and VPI10463 biofilms using the XTT (2-methyloxy-4-nitro-5-sulfophen​yl]-2H-tetrazolium-5-carboxanilide)-menadione reduction assay (Figs. 1, 2, 3, 4 and 5). Against 20291 R027, it was found that each of the antimicrobials, with the exception of vancomycin and teicoplanin, were effective at decreasing the viability of the biofilms at 4x the MIC concentration (Fig. 1). At 2x the MIC concentrations, the antimicrobials used were less potent at reducing the R027 biofilm cell viability, with teicoplanin, vancomycin and tigecycline being the least effective (Fig. 1). *Clostridium difficile* DPC6350 was determined to be a relatively strong biofilm former and it was found that teicoplanin, tigecycline, vancomycin and thuricin CD were particularly effective at targeting biofilms of this strain at both 4x and 2x MIC concentrations (all P < 0.002)(Fig. 2).

*C. difficile* Liv022 R106 was a relatively weaker biofilm former compared to strains 20291 and DPC6350 (Fig. 3). It was found that thuricin CD at 4x MIC was effective at reducing the viability of the biofilm relative to the untreated control (P < 0.05). We also observed that although there was a minor reduction in R106 biofilm viability after treatment with nitazoxanide, vancomycin, teicoplanin, tigecycline and rifampicin at 4x MIC, these reductions were not statistically significant (all P > 0.05) (Fig. 3).

*Clostridium difficile* TL178 R002 was found to be a weak biofilm former, compared to strains R027 and DPC6350 (Fig. 4). Against TL178 R002, it was apparent that none of the antibiotics were effective at reducing the biofilm viability when used independently, at concentrations of 4x MIC or 2x MIC. Thuricin CD at 4x MIC elicited a minor reduction in R002 biofilms relative to the untreated control but this difference was not significant (P > 0.05) (Fig. 4).

Finally, *C. difficile* VPI10463 was found to be the weakest biofilm former amongst the strains tested under our incubation conditions (Fig. 5). Thuricin CD at 4x MIC and 2x MIC elicited a reduction in the viability of VPI10463 biofilms (both P < 0.05). Amongst the antibiotics, 4x MIC nitazoxanide was the only antibiotic that led to a significant reduction in VPI10463 biofilms (P < 0.05), whereas the other antibiotics were unable to do so (Fig. 5).

### Efficacy of antimicrobials in paired combinations against *C. difficile* biofilms

In addition to assessing the potencies of the above-mentioned antimicrobials used independently against 20291 R027 biofilms, we also combined the sactibiotic thuricin CD at 2x the MIC concentration with 2x MIC of each of the antibiotics vancomycin, rifampicin, teicoplanin, tigecycline or nitazoxanide (Figs. 1, 2, 3, 4 and 5). Combinations of 2x MIC of thuricin CD and 2x MIC of rifampicin against *C. difficile* R027 biofilms appeared to be potent, as the combination led to statistically significant differences in terms of reduction of the biofilm, relative to 2x MIC of the two antimicrobials used independently (P < 0.0001 and 0.005, respectively) and relative to the untreated control (P < 0.0001)(Fig. 1). Similarly, there were statistically significant differences between the combination of 2x MIC thuricin CD and 2x MIC tigecycline, relative to 2x MIC of the antimicrobials used independently (P < 0.05 and 0.005, respectively) and relative to the untreated control (P < 0.0001), although the difference was less pronounced than that of thuricin CD-rifampicin combinations (Fig. 1). Thuricin CD-teicoplanin combinations resulted in similar effects as those resulting from the thuricin CD-tigecycline combinations described above (Fig. 1).With respect to thuricin CD-vancomycin combinations against R027 biofilms, the decrease in viability of the biofilm relative to 2x MIC thuricin CD was not statistically significant (P > 0.05) but was significant when compared to 2x MIC vancomycin used independently (P < 0.05) and relative to the untreated control (P < 0.005). Interestingly, thuricin CD-nitazoxanide combinations against *C. difficile* R027 biofilms resulted in an apparent antagonistic effect, as 2x MIC of thuricin CD combined with 2x MIC of nitazoxanide was ineffective at decreasing the viability of the biofilm, relative to the use of 2x MIC thuricin CD (P < 0.005) or 2x MIC nitazoxanide alone (P < 0.005). We sought to assess whether higher concentrations of thuricin CD (8x MIC) combined with higher concentrations of nitazoxanide (8x MIC) resulted in a similar apparently antagonistic effect as seen with 2x thuricin CD-2x nitazoxanide combinations against R027 biofilms. However, we found that 8x MIC thuricin CD-8x MIC nitazoxanide combinations were potent against R027 biofilms, with a marked reduction in viability, relative to the untreated control (P < 0.001)(Fig. 1).

Against biofilms of strain DPC6350, we determined that 2x MIC thuricin CD combined with 2x MIC of each of the five antibiotics elicited a decrease in biofilm viability (all P < 0.001) (Fig. 2). Combinations of 8x MIC thuricin CD combined with 8x MIC nitazoxanide were also effective against DPC6350 biofilms, relative to the untreated control (P < 0.001). As was the case with 20291 biofilms, thuricin CD-rifampicin combinations appeared to be the most effective at targeting DPC6350 biofilms (Fig. 2).

With respect to the antimicrobial combinations against *C. difficile* Liv022 R106 biofilms, it was found that none of the antibiotics combined with thuricin CD led to statistically significant reductions in biofilm viability, when compared to the untreated control (all P > 0.05)(Fig. 3). In contrast to strains 20291 and DPC6350, the most effective combination against Liv022 R106 biofilms appeared to be 2x MIC thuricin CD combined with 2x MIC nitazoxanide and the least effective was 2x MIC thuricin CD with 2x MIC rifampicin. Overall, there were no statistically significant improvements in the potency of the antibiotic combinations with thuricin CD against Liv022 R106 biofilms, when compared to the potency of 2x thuricin CD used independently (all P > 0.05). High concentrations of thuricin CD combined with high concentrations of nitazoxanide (both 8x MIC) were potent at decreasing the viability of Liv022 R106 biofilms, relative to the untreated control (P < 0.005) (Fig. 3).

Similar to strain Liv022 R106, the combinations of 2x MIC of the different antibiotics with 2x MIC thuricin CD failed to result in any reductions in TL178 R002 biofilm viability, relative to the untreated control (all P > 0.05)(Fig. 4). Even high concentrations of thuricin CD (8x MIC) combined with high concentrations of nitazoxanide (8x MIC) was ineffective at reducing TL178 R002 biofilm viability. Furthermore, there was no improvement in terms of potency against TL178 R002 biofilms, when 2x MIC thuricin CD was combined with 2x MIC of each of the antibiotics, compared to 2x MIC thuricin CD used on its own (Fig. 4). Finally, against strain VPI10463, which was the weakest biofilm former evaluated in this study, it was determined that thuricin CD-rifampicin combinations were the most effective against biofilms of this strain (P < 0.005), relative to the untreated control (Fig. 5).

## Discussion

The aim of the study was to evaluate the efficacy of one bacteriocin (thuricin CD) and 5 antibiotics (teicoplanin, tigecycline, vancomycin, rifampicin and nitazoxanide) when used independently and when combined at low concentrations together against *C. difficile* biofilms, as well as against planktonic cells of the same strains. As antibiotics are expensive to use in clinical settings, we attempted to find alternative antimicrobial combinations which work well together at relatively low concentrations (2x MIC each), in an attempt to diminish the viability of such biofilms. Furthermore, since biofilms often show resistance to antibiotics used on their own, our objective was to search for effective antimicrobial combinations that could potentiate each other’s effects to circumvent these problems of antimicrobial resistance. We postulated that using antimicrobial combinations at low concentrations would provide insights into effective therapeutic options with a view to targeting *C. difficile* biofilms.

It was particularly noteworthy there were differences amongst *C. difficile* strains in their abilities to form strongly adherent biofilms and variations in the sensitivities of biofilms of these different *C. difficile* strains that we tested to the different antibiotics used either independently or in paired combinations. Various factors are likely to be involved in governing the strengths of biofilms formed, including the presence of glucose, sporulation and even sub-lethal doses of antibiotics [3, 5, 19, 20]. This potential stimulation of *C. difficile* biofilm formation by sub-lethal concentrations of antibiotics may occur in treatment regimens involving pulsed or tapered dosing of antibiotics against CDI, whereby antibiotics are likely to be present at sub-lethal doses and thus can contribute to treatment failure [3, 19]. *Clostridium difficile* R20291 R027 and DPC6350 were the strongest biofilm formers amongst the strains we tested in this study. *C. difficile* R20291 R027 has also previously been shown to be a relatively strong biofilm former in other studies [2, 3, 5]. A stronger ability to form adherent biofilms may be a contributing factor involved in greater degrees of colonization of this epidemic-associated strain in the gut [3, 5].

With respect to the antimicrobials used independently in our study, the glycopeptide vancomycin was found to be ineffective against biofilms of four of the five strains we assessed. Similar to our findings, Dapa and co-workers had also previously reported that *C. difficile* biofilms display attenuated sensitivity to vancomycin [5**].** Indeed, biofilms are generally more resistant to antibiotics and studies by Semenyuk et al. had reported that *C. difficile* biofilms also exhibit increased resistance to the antibiotic metronidazole, relative to *C. difficile* planktonic cells grown in liquid broth media [21]. The study by Dapa et al. indicates that the structure and organisation of the matrix in *C. difficile* biofilms is likely to play a role in mediating resistance to antibiotics, as such matrices can prevent penetration of the antimicrobial reaching the bacteria within [5, 22]. In addition, the physiological state of the bacteria present in the biofilm as well as the presence of persister cells are likely to have a role in attenuated sensitivity to antibiotics [23]. Interestingly, the semi-synthetic glycopeptide, teicoplanin, was found to be effective only against biofilms of *C. difficile* strain DPC6350, while being ineffective against the other strains in our study. Thus, it appears that the glycopeptide group of antibiotics can be ineffective at targeting biofilms of a number of *C. difficile* strains. The thiazolide antimicrobial, nitazoxanide, was effective at reducing the biofilm viability of three of the five strains we tested in this study and it was determined that tigecycline (a member of the glycylcycline group of antibiotics) was potent against 20291 R027 and DPC6350, while being ineffective against biofilms of strains TL178 R002, Liv022 R106 and VPI10463. It may be the case that tigecycline has a greater ability to target strongly-adherent biofilms, such as those formed by R027, DPC6350, and is ineffective against weakly-adherent biofilms. Similar to tigecycline, rifampicin (belonging to the rifamycin group of antibiotics) also exhibited more potency against the stronger biofilms of R027 and DPC6350, while only being marginally effective against Liv022 R106 biofilms and ineffective against the weak biofilms of TL178 R002 and VPI10463. It is plausible that altered growth rates of cells within these *C. difficile* biofilms caused by genetic mutations leads to attenuated sensitivity of certain strains to teicoplanin, tigecycline and rifampicin. Thus, it is clear that biofilms of different *C. difficile* strains display varying degrees of sensitivity to the different antibiotics when used on their own but the precise mechanisms of sensitivity remain largely unknown.

With respect to antimicrobial combinations used in this study, variations in sensitivities of biofilms of different strains to the antimicrobial combinations were also apparent. The most striking difference was that while thuricin CD combined with either teicoplanin, tigecycline, vancomycin or rifampicin was effective against stronger biofilms of strains 20291 R027 and DPC6350, such combinations were largely ineffective against the relatively weaker biofilms of TL178 R002, Liv022 R106 and VPI10463. Interestingly, 2x thuricin CD-2x nitazoxanide combinations were particularly ineffective against R027 biofilms. It may be the case that the addition of thuricin CD to nitazoxanide at low concentrations somehow interferes with the ability of nitazoxanide to target the pyruvate:ferredoxin oxidoreductase system in *C. difficile* R027 at such concentrations. Alternatively, it could be the case that nitazoxanide prevents thuricin CD from reaching a target receptor in *C. difficile*, thus leading to a lack of efficacy against *C. difficile* R027 biofilms when combined together, resulting in apparent antagonistic effects at these concentrations. Furthermore, while higher concentrations of these antimicrobials combined (8x MIC thuricin CD with 8x MIC nitazoxanide) were effective against biofilms of R027, DPC6350 and Liv022 R106, this combination at higher concentrations was still ineffective against the weakest biofilms of TL178 R002 and VPI10463. Thus, it is apparent that there are strain-specific as well as concentration-dependent variations with regards to sensitivities of *C. difficile* biofilms to different antimicrobials and antimicrobial combinations. Since the XTT-menadione reduction assay determines the level of viability of *C. difficile* biofilms, it provides insights into the amount of a biofilm that is still metabolically active, subsequent to antimicrobial challenges and thus is likely to provide a realistic picture regarding the potency of antimicrobials against biofilms in vivo [24–27]. The precise mechanism of action involved in thuricin CD-nitazoxanide against *C. difficile* planktonic cells and biofilms remains unclear however and merits further investigation in future studies.

With respect to utilising FIC values against *C. difficile* planktonic cells as a predictor of effective combinations against biofilms of the same strains, the partial synergistic and additive effects seen with thuricin CD-teicoplanin, thuricin CD-rifampicin and thuricin CD-tigecycline combinations against *C. difficile* R027 planktonic cells are consistent with additive effects against R027 biofilms (Fig. 1; Table 2). Combinations of the sactibiotic thuricin CD with vancomycin, rifampicin, tigecycline and teicoplanin may enable one of the antimicrobials to gain access through the complex matrix and allow the antimicrobial to exert its killing effect on the R027 biofilm, thereby leading to a reduction in biofilm viability. In contrast, there doesn’t appear to be a correlation between the additive effects (FIC 1.0) obtained with thuricin CD-vancomycin combinations against planktonic cells of Liv022 R106 and the combination against biofilms of the same strain. In addition, even though thuricin CD combined with rifampicin resulted in indifferent effects (FIC 1.01–2) against planktonic cells of DPC6350, it is apparent that this combination displayed ameliorated potency against DPC6350 biofilms, compared to either 2x MIC thuricin CD or 2x MIC rifampicin. Finally, while thuricin CD-nitazoxanide combinations resulted in additive effects (FIC 1.0) against planktonic cells of DPC6350, combinations of 2x MIC thuricin CD with 2x MIC nitazoxanide did not exhibit enhanced activity when compared to 2x MIC thuricin CD used independently against biofilms of this strain (P > 0.05). Similar trends were noted against planktonic cells and biofilms of strains TL178 R002 and VPI10463. The variations between planktonic cells and biofilms could be due to differences in the mechanisms of action of thuricin CD-nitazoxanide combinations against planktonic cells, as opposed to the two antimicrobials targeting a complex multi-layered matrix that exists in a *C. difficile* biofilm.

## Conclusions

In conclusion, this is the first study assessing the antimicrobial effect of a sactibiotic bacteriocin and the antibiotics nitazoxanide, tigecycline and teicoplanin against *C. difficile* biofilms. Overall, it is encouraging to note from this study that a number of *C. difficile* strains have a relatively weak ability to form strongly adherent biofilms, in comparison to other pathogens such as *Streptococcus mutans*, *Staphylococcus aureus* and *Pseudomonas aeruginosa,* which are notorious biofilm producers [28–30]. It is also encouraging to note that four of the five antimicrobial combinations we tested (with the exception of thuricin CD-nitazoxanide) appeared to be highly effective against two of the stronger *C. difficile* biofilm formers, 20291 R027 and DPC6350. It is apparent from this study that, not only are there variations between different *C. difficile* strains in their abilities to form biofilms, there are also variations in terms of sensitivities of biofilms of different strains to several antimicrobial treatment options. Such variations seen with regards to the potencies of the different antimicrobials/antimicrobial combinations against these different strains may be due to the relative strengths of the biofilms that the antimicrobials target. Furthermore, the mechanism of action involved in effective antimicrobial combinatorial therapy is likely to be different against planktonic cells, compared to cells in biofilms. While it is clear that much remains to be elucidated with respect to the precise mechanisms governing antimicrobial sensitivity and antimicrobial resistance within *C. difficile* biofilms, overall, this study could form the basis for the development of successful antimicrobial combination therapy strategies, with a view to targeting *C. difficile* biofilms. The findings presented in this study could have implications with regards to reducing the recurrence rates of CDI, particularly in cases where a strong *C. difficile* biofilm former is contributing to increased recurrence rates.

## Abbreviations

CDI: *Clostridium difficile* infection
EPS: extracellular polymeric substance
MIC: minimum inhibitory concentration
XTT: (2,3-bis[2-methyloxy-4-nitro-5-sulphophenyl]-2H-tetrazolium-5-carboxanilide)
cfu: colony forming unit
BHI: brain heart infusion
FIC: fractional inhibitory concentration
OD: optical density
